# Mitophagy-Related Gene Signature for Prediction Prognosis, Immune Scenery, Mutation, and Chemotherapy Response in Pancreatic Cancer

**DOI:** 10.3389/fcell.2021.802528

**Published:** 2022-02-07

**Authors:** Zewei Zhuo, Hanying Lin, Jun Liang, Pengyue Ma, Jingwei Li, Lin Huang, Lishan Chen, Hongwei Yang, Yang Bai, Weihong Sha

**Affiliations:** ^1^ School of Bioscience and Bioengineering, South China University of Technology, Guangzhou, China; ^2^ Department of Gastroenterology, Guangdong Provincial People’s Hospital, Guangdong Academy of Medical Sciences, Guangzhou, China; ^3^ Department of Endocrinology, The First People’s Hospital of Zhaoqing, Zhaoqing, China; ^4^ Department of Geriatric Intensive Care Unit, Guangdong Provincial Geriatrics Institute, Guangdong Provincial People's Hospital, Guangdong Academy of Medical Sciences, Guangzhou, People’s Republic of China; ^5^ Department of Nephrology, The Second Affiliated Hospital of Zhengzhou University, Zhengzhou, China; ^6^ School of Clinical Medicine, Guangdong Pharmaceutical University, Guangzhou, China; ^7^ Department of Obstetrics and Gynecology, The First Affiliated Hospital of Guangzhou Medical University, Guangzhou, China; ^8^ Guangdong Provincial Key Laboratory of Gastroenterology, Department of Gastroenterology, Institute of Gastroenterology of Guangdong Province, Nanfang Hospital, Southern Medical University, Guangzhou, China

**Keywords:** mitophagy, signature, prognosis, immune scenery, mutation, chemotherapy response, pancreatic cancer

## Abstract

Mitophagy is a conserved cellular process that plays a vital role in maintaining cellular homeostasis by selectively removing dysfunctional mitochondria. Notwithstanding that growing evidence suggests that mitophagy is implicated in pancreatic tumorigenesis, the effect of mitophagy-related genes on pancreatic cancer (PC) prognosis and therapeutic response remains largely unknown. In this study, we sought to construct a mitophagy-related gene signature and assessed its ability to predict the survival, immune activity, mutation status, and chemotherapy response of PC patients. During the screening process, we identified three mitophagy-related genes (PRKN, SRC, VDAC1) from The Cancer Genome Atlas (TCGA) cohort and a 3-gene signature was established. The prognostic model was validated using an International Cancer Genome Consortium (ICGC) cohort and two Gene Expression Omnibus (GEO) cohorts. According to the median risk score, PC patients were divided into high and low-risk groups, and the high-risk group correlated with worse survival in the four cohorts. The risk score was then identified as an independent prognostic predictor, and a predictive nomogram was constructed to guide clinical decision-making. Remarkably, enhanced immunosuppressive levels and higher mutation rates were observed in patients from the high-risk group, which may account for their poor survival. Furthermore, we found that high-risk patients were more sensitive to paclitaxel and erlotinib. In conclusion, a mitophagy-related gene signature is a novel prognostic model that can be used as a predictive indicator and allows prognostic stratification of PC patients.

## Introduction

Pancreatic cancer (PC) is a devastating digestive tract malignancy accounting for 4.7% of cancer-related deaths, and could reportedly become the third leading cause of cancer death by 2025 (Sung et al., 2021). High heterogeneity, difficult early diagnosis, and limited efficacy account for the unfavorable prognosis of PC (Park et al., 2021). Emerging evidence has reported that targeted therapy based on genetic testing may provide an effective treatment option to overcome these drawbacks during PC therapy (Dokus et al., 2020). Nevertheless, most gene expression variations remain poorly characterized due to their complex molecular subtyping and tumor heterogeneity in PC patients, restraining their clinical translation (Hosein et al., 2020; Giuliani et al., 2021; Wang et al., 2021). Accordingly, a novel biomarker is urgently required for prognostic stratification and therapeutic purposes to enhance the outcome of the PC patients.

Mitophagy is a mitochondrial quality control mechanism responsible for metabolic remodeling within tumor cells and for regulating interactions among tumor cells (Li et al., 2021). Functionally activated by hypoxia and metabolic stress, mitophagy enhances tumor cell survival by removing redundant mitochondria and reducing oxygen consumption (Vara-Perez et al., 2019). A previous study demonstrated that loss of mitophagy negatively impacts pancreatic cancer stem cells stemness, impairing their tumorigenic capacity (Alcala et al., 2020). Recently, mitophagy-related genes such as PINK1/PRKN and BNIP3L have also been documented in pancreatic tumorigenesis (Humpton et al., 2019; Zhao et al., 2019), and are potential targets for PC treatment (Li et al., 2021). Therefore, a comprehensive analysis of key modulator of mitophagy involved in PC progression and prognosis can guide clinical decision-making and provide more therapeutic options for PC patients.

Accumulating evidence shows that tumor development and progression largely depend on their complex microenvironment which they reside in, including tumor cells and their surrounding immune cells, fibroblasts, and endothelial cells (Zhang K. et al., 2013). Some researchers suggest that immune score can be performed as part of cancer staging system based on the traditional TNM stage system to further improve the assessment of overall prognosis (Angell et al., 2020). Recently, immunoscore-based cancer classification has been established in colon cancer, and results indicated that the prognostic value of infiltration of adaptive immune cells is superior to that of current classical tumor infiltration (TNM stage) (Pages et al., 2018). Therefore, introduction of immune scoring system will further contribute to provide novel insights into PC stratification and prognosis predictions.

Considering the significant value of mitophagy coupled with immunoscore-based cancer classification in PC, we identified differentially expressed mitophagy-related genes between the high and low-immune subtypes in PC patients and then developed a mitophagy-related prognostic signature using The Cancer Genome Atlas (TCGA) cohort. The prognostic efficacy of the novel model was validated using International Cancer Genome Consortium (ICGC) and Gene Expression Omnibus (GEO) cohorts. Moreover, we also investigated the correlation of mitophagy-related prognostic signature with immune infiltration levels, mutations, and chemotherapy response, confirming the predictive and prognostic value of the novel model.

## Materials and Methods

### Data Collection

Transcriptome sequencing data [Fragments Per kilobase of transcript per Million mapped reads (FPKM) normalized], mutation data and corresponding clinical information of PC were obtained from TCGA database and used as the training set (https://tcga-data.nci.nih.gov/tcga/). Validation cohorts were downloaded from the ICGC database (https://dcc.icgc.org) and the GEO database (https://www.ncbi.nlm.nih.gov/geo). In total, 176 samples from TCGA-Pancreatic Adenocarcinoma (TCGA-PAAD), 79 samples from ICGC-Pancreatic Cancer-Australia (ICGC-PACA-AU), 41 samples from GSE28735 (Zhang G. et al., 2013), and 62 samples from GSE62452 (Yang et al., 2016) were collected for analysis. The transcript sequencing data of TCGA, read counts data of ICGC, and series matrixes data of GSE28735, GSE62452 were processed by “log2 (data +1).“, which were conducted by the R package “limma” (Ritchie et al., 2015). Additionally, the immune scores of PC patients were obtained from the ESTIMATE database (https://bioinformatics.mdanderson.org/estimate/disease.html). 29 mitophagy-related genes were downloaded from the Pathway Unification database (https://pathcards.genecards.org/), listed in Supplementary Table S1.

### Differentially Expressed Genes (DEGs) Screening

PC samples from the TCGA cohort were classified into high and low-immune groups based on the median immune score. Next, 29 MRGs expression profiles were extracted from the whole expression data in TCGA cohort, and the R package “pheatmap” was used to generate a heatmap. Significant DEGs were identified between two immune groups with a false discovery rate threshold (FDR) of <0.05. We applied R packages “reshape2” and “ggpubr” to draw boxplots of DEGs expression. The correlation network of DEGs was generated by R package “igraph” and the Protein-Protein Interaction (PPI) network of DEGs was constructed by the search tool for retrieval of interacting genes (STRING) database (http://string-db.org).

### Tumor Classification Based on MRGs

To further explore the relationship between 29 MRGs and PC subtypes, we used the R package “ConsensusClusterPlus” for consistency analysis. Kaplan-Meier survival analysis of the different subtypes was performed by R package “survival” and “survminer”. The R package “pheatmap” was applied to draw the heatmap.

### Construction and Validation of A Mitophagy-Related Gene Signature

Univariate Cox regression analysis was used to select survival-associated genes. Then multivariate Cox regression analysis was applied to screen for genes independently related to survival. To further narrow down the candidate genes, we applied the least absolute shrinkage and selection operator (LASSO) algorithm to prevent model overfitting. A risk score was calculated by Lasso regression coefficients, and the formula used was as follows:

$$Risk\;score=\overset{n}{\underset{i=1}{\sum }}Xi+Yi$$

Where X represents coefficients and Y is gene expression level. Based on the median risk score, PC samples were divided into high and low-risk groups. Kaplan-Meier analysis was used to analyze the survival difference between two risk groups. Principal component analysis (PCA) was conducted using the “prcomp” function of the “stats” package in R. To assess the performance of the prognostic model, area under the ROC curve (AUC) analysis was conducted using the R package “timeROC”. In both validation cohorts (ICGC-PACA-AU, GSE28735, and GSE62452), the risk score was calculated using the same formula and the methods mentioned above were used to validate the performance of the risk signature.

### Analyses of Signature Genes

To further explore the expression and clinical correlation of signature genes between the two risk groups, we applied the R package “pheatmap” to generate a heatmap. The survival analysis of signature genes was conducted using the R package “survival”. The expression of signature genes was validated in the Human Protein Atlas (HPA) database (www.proteinatlas.org). Next, we generated a heatmap of the relationship between signature genes and immune cells using R package “pheatmap”. The correlation between the abundance of immune infiltrating cells and signature genes was evaluated using the Tumor Immune Estimation Resource (TIMER) database.

### Establishment of Nomogram

Clinical characteristics were extracted from the TCGA cohort, and then univariate and multivariate Cox regression analyses were performed to identify the independent prognostic factors. Based on the results of the multivariate analysis, we applied the R package “rms” to create a nomogram for guiding clinical decision-making. The C-index, calibration curve, and AUC were used to assess the predictive accuracy of the nomogram.

### Functional Enrichment Analyses

Using the criteria |log_2_FC| ≥ 1 and FDR <0.05, we screened for DEGs of the two risk groups. The functions of DEGs were annotated by Gene Ontology (GO) and Kyoto Encyclopedia of Genes and Genomes (KEGG) using R packages “clusterProfiler” and “org.Hs.eg.db”. Single-sample gene set enrichment analysis (ssGSEA) was used to calculate the score of immune infiltrating cells and the activity of pathways.

### Assessment of Tumor Immune Cell Infiltration

To further explore the immune infiltration cells associated with our prognostic signature, Spearman correlation analysis was conducted between the risk score and the expression of six immune infiltration cells (B cell, CD4^+^ T cell, CD8^+^ T cell, neutrophil, macrophage, and myeloid dendritic cell) by R package “ggstatsplot”. The composition and differences in the 22 kinds of tumor-infiltrating immune cells between two risk groups were calculated by the CIBERSORT algorithm.

### Immune Checkpoint Analysis

The expression level of eight immune-checkpoint genes [CD274, cytotoxic T-lymphocyte associated protein 4 (CTLA4), hepatitis A virus cellular receptor 2 (HAVCR2), lymphocyte activating 3 (LAG3), programmed cell death 1 (PDCD1), programmed cell death 1 ligand 2 (PDCD1LG2), sialic acid binding Ig like lectin 15 (SIGLEC15), and T cell immunoreceptor with Ig and ITIM domains (TIGIT)] in the two risk subgroups were determined using the R packages “ggplot2” and “reshape2”.

### Mutation Status Analysis

We extracted the sample mutation data in the TCGA cohort. Then the R package “maftools” was used to analyze the mutation status between two risk subgroups.

### Drug Susceptibility Analysis

Half-maximal inhibitory concentration (IC50) is an important indicator for evaluating drug effects or sample treatment response, where the lower IC50 indicates a high anti-tumor capacity (Gomaa, 2017). To assess the chemotherapeutic sensitivity of the prognostic model, we put our predictive signature into the currently and the largest publicly available pharmacogenomics database [Genomics of Drug Sensitivity in Cancer (GDSC), https://www.cancerrxgene.org/] for training. The prediction process was conducted using the R package “pRRophetic” and the IC50 value estimate of chemotherapeutic drugs was estimated by ridge regression. All parameters were set by the default values, and duplicate gene expression was summarized as mean value (Tang et al., 2020).

### Statistical Analysis

All statistical analyses were performed by R software. Comparisons of two groups were conducted by the Student’s t-test or the Wilcoxon test. The Kruskal-Wallis test was used to compare three groups or above. Additionally, Spearman correlation was used to assess the correlation between two continuous variables. *p* < 0.05 was statistically significant.

## Results

### Identification of DEGs Between Two Immune Subgroups

**FIGURE 1:**
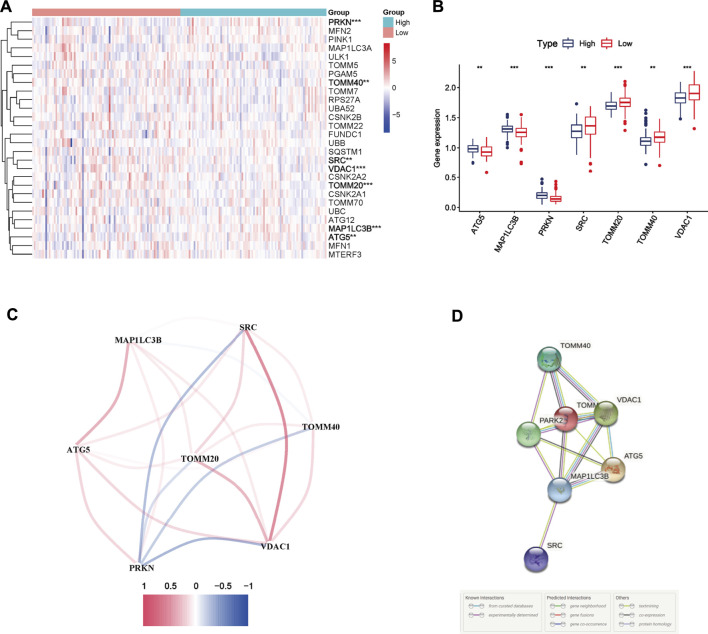
Mitophagy-related differentially expressed genes (DEGs) between two immune subgroups in The Cancer Genome Atlas (TCGA) cohort **(A)** Heatmap of 29 mitophagy-related genes expression profiles between the high and low-immune group of pancreatic cancer patients from TCGA cohort. * represents *p* < 0.05; ** represents *p* < 0.01; *** represents *p* < 0.001 **(B)** Boxplots of the expression of DEGs **(C)** Correlation network of DEGs. Red represents positive correlations while blue represents negative correlations **(D)** Protein-protein interaction (PPI) network of DEGs.

According to the median immune score, PC samples were divided into two immune subgroups. Seven MRGs were differentially expressed between the high and low-immune groups (all FDR <0.05, Figures 1A,B). Among them, three genes [autophagy related 5 (ATG5), microtubule associated protein 1 light chain three beta (MAP1LC3B), parkin RBR E3 ubiquitin protein ligase (PRKN)] were downregulated while four other genes [SRC, translocase of outer mitochondrial membrane 20 (TOMM20), translocase of outer mitochondrial membrane 40 (TOMM40), voltage dependent anion channel 1 (VDAC1)] were upregulated in high-immune group. The correlation network of DEGs was presented in Figure 1C, and different colors reflect different correlation coefficients. Furthermore, the PPI network was constructed to identify the interactions of these mitophagy-related DEGs, and the results of PPI network analysis indicated that TOMM20 was a hub gene (Figure 1D).

### Tumor Classification Based on MRGs

**FIGURE 2:**
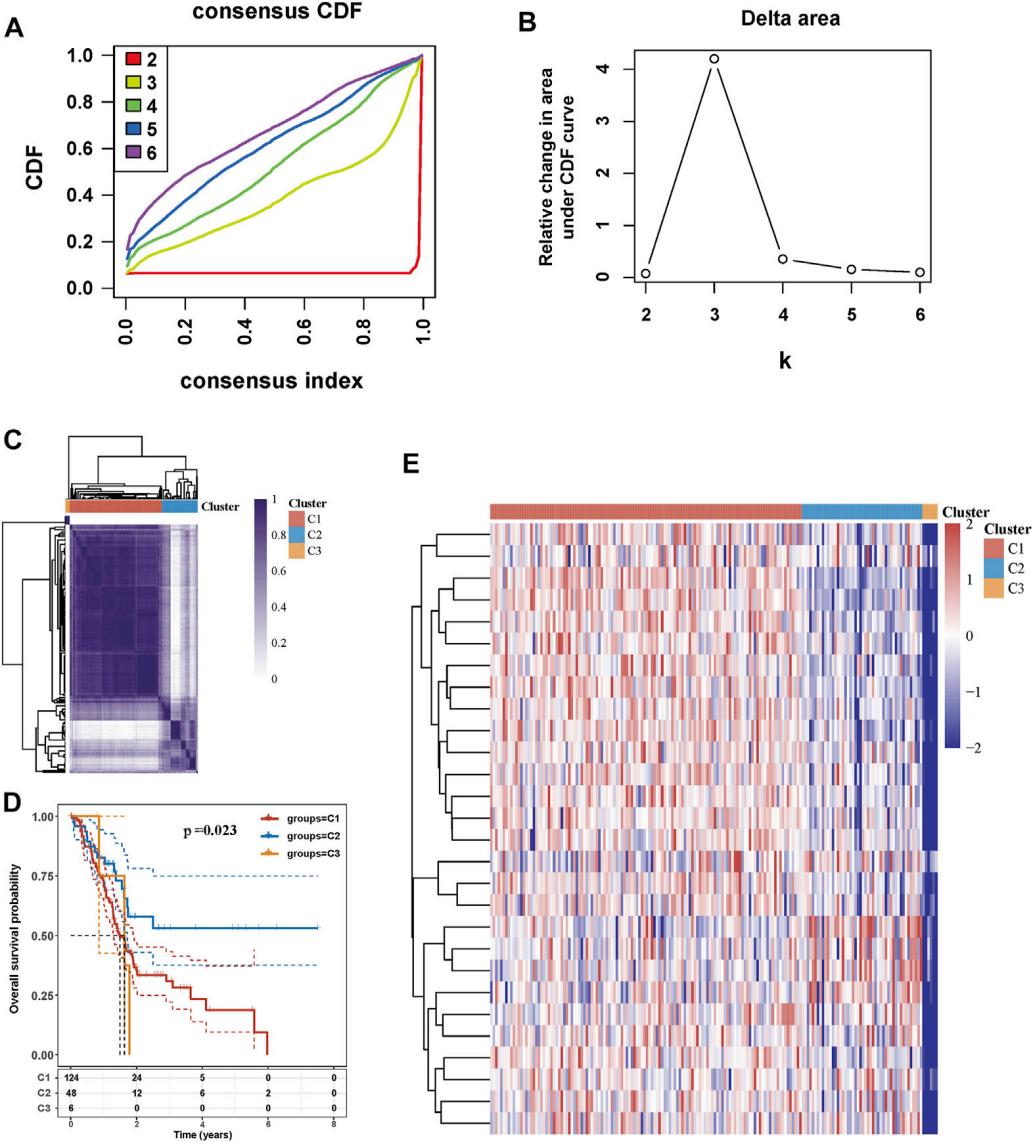
Tumor classification based on mitophagy-related genes **(A)** Cumulative distribution function (CDF) curves **(B)** Delta area curve of consensus clustering **(C)** Consensus clustering matrix (K = 3) **(D)** Kaplan-Meier survival analysis of the three subgroups **(E)** Heatmap of mitophagy-related gene expression in the three subtypes.

Consensus clustering analysis was conducted to assess the effect of MRGs on PC samples. Based on the results of the Cumulative distribution function (CDF) curves and relative change in the area under the CDF curve, as shown in Figure 2A,B, K = 3 was determined as the optimal cluster number, which corresponds to the largest number of clusters that induced the smallest incremental change in the area under the CDF curves while keeping the maximal consensus within clusters. The result of Figure 2C showed that PC samples could be divided into three clusters based on mitophagy-related genes. PC patients from the TCGA cohort were grouped into three clusters (N = 124, 48, and 6, respectively), and survival among the three subtypes was significantly different (*p* = 0.023, Figure 2D). The gene expression profile between the three clusters is shown in Figure 2E.

### Establishment and Validation of the three Mitophagy-Related Gene Signature

**FIGURE 3:**
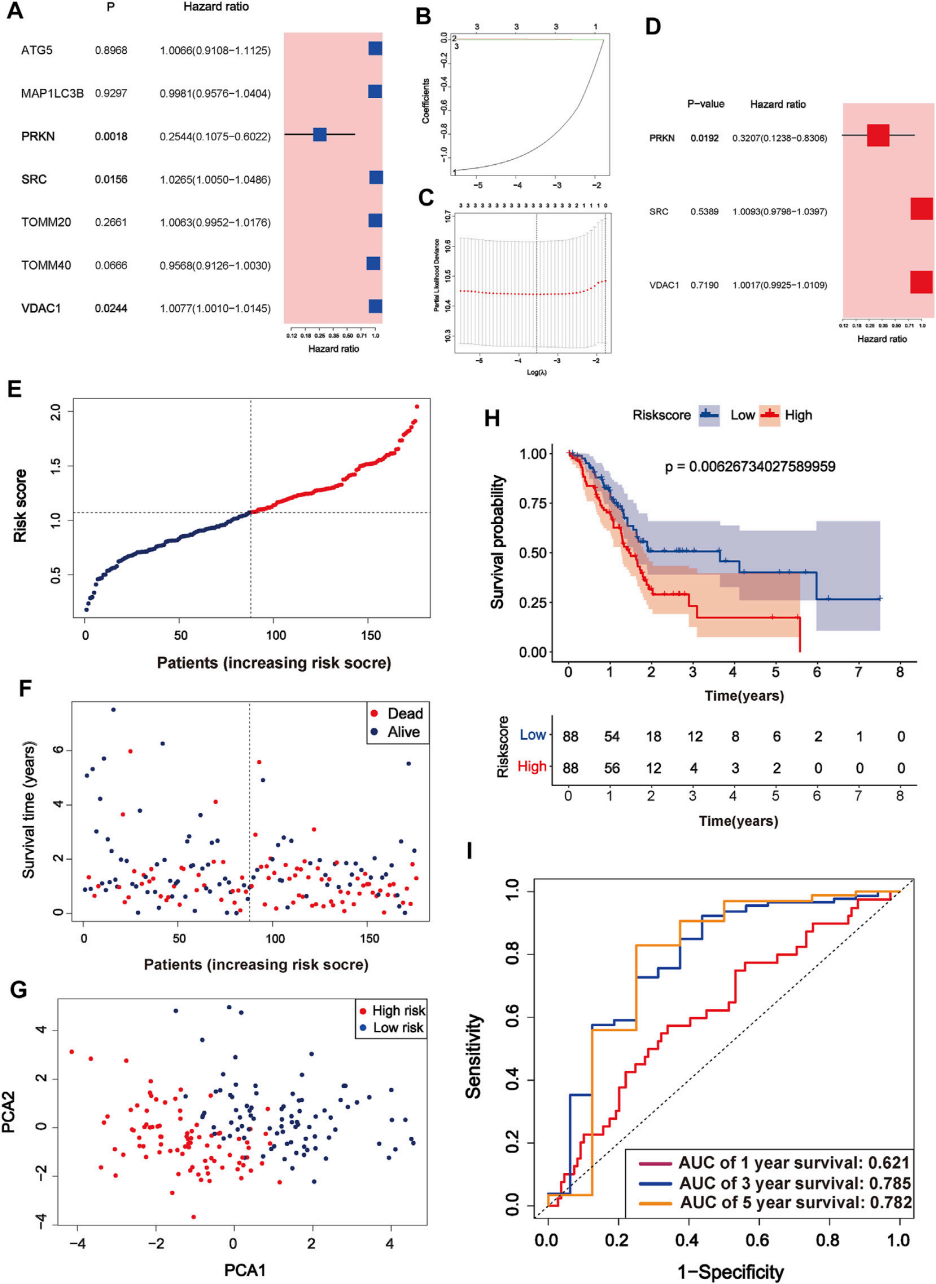
Identification of mitophagy-related gene signature in TCGA cohort **(A)** A forest plot of prognosis-related genes after univariate Cox regression analysis **(B)** The process of least absolute shrinkage and selection operator (LASSO) variables screening **(C)** Cross-validation of candidate genes based on the minimum lambda value **(D)** A forest plot of independent prognosis-related genes using multivariate Cox regression analysis **(E)** Distribution of risk score for each PC sample in the TCGA cohort **(F)** Survival time and status of each PC sample based on the risk score **(G)** Principal component analysis (PCA) of the 3-gene signature **(H)** Survival analysis between the two risk subgroups **(I)** ROC curve of the 3-gene signature.

After univariate Cox regression analysis, three MRGs were determined as prognosis-related genes from the TCGA cohort. SRC and VDAC1 were risk genes (HR > 1), whereas PRKN was a protective gene (HR < 1) (Figure 3A). The LASSO regression further narrowed down the candidate genes, and a 3-gene signature was eventually established based on the optimum λ value 0.02895928 (Figures 3B,C). Multivariate Cox regression further screened the prognosis-associated genes and PRKN was considered an independent prognosis gene (*p* = 0.0192, Figure 3D). The risk score was calculated as follows:

$$Risk\;score=\left(-0.929609894\right)*Expression^{PRKN}+0.006652335*Expression^{SRC}+0.001093884*Expression^{VDAC1}$$

**FIGURE 4:**
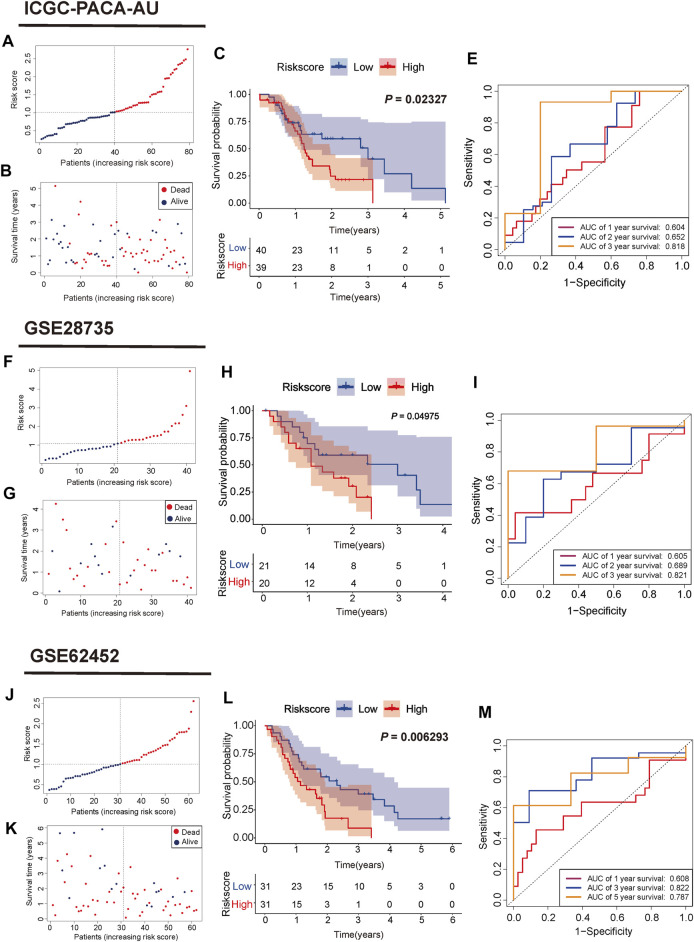
Validation of the mitophagy-related gene signature in ICGC, GSE28735, GSE62452 cohorts **(A,F,J)** Distribution of risk score for each PC sample **(B,G,K)** Survival time and status of each PC sample based on the risk score **(C,H,L)** Survival analysis between the two risk subgroups **(E, I,M)** ROC curve of the 3-gene signature.

According to the median risk score, PC samples were divided into high (n = 88) and low-risk groups (n = 88) and patients with higher risk scores were associated with a greater risk of death and shorter survival time (Figure 3E,F). PCA showed a clear distinction between the high and low-risk groups (Figure 3G). Survival curves indicated that high-risk patients had worse survival than low-risk patients (*p* = 0.006267, Figure 3H). The three and 5-years survival AUC value in the TCGA cohort was 0.785 and 0.782, respectively (Figure 3I). External validation sets showed high prediction accuracy of our 3-gene signature. Risk score distribution of PC samples and their survival status in the cohorts were presented in Figure 4A,B,F,G,J,K. Consistent with the result in the TCGA cohort, high-risk patients had significantly poorer survival than low-risk patients in the three cohorts (ICGC-PACA-AU, *p* = 0.02327, Figure 4C; GSE28735, *p* = 0.04975, Figure 4H; GSE62452, *p* = 0.0062, Figure 4L). Furthermore, the AUC values of three validation cohorts demonstrated good predictive ability and robustness for survival at 3-years (ICGC-PACA-AU, AUC = 0.818, Figure 4E; GSE28735, AUC = 0.821, Figure 4I; GSE62452, AUC = 0.822, Figure 4M), indicating that our 3-gene signature could reliably predict PC patient survival.

### Three Signature Genes Were Associated With Survival and Immune Activity

**FIGURE 5:**
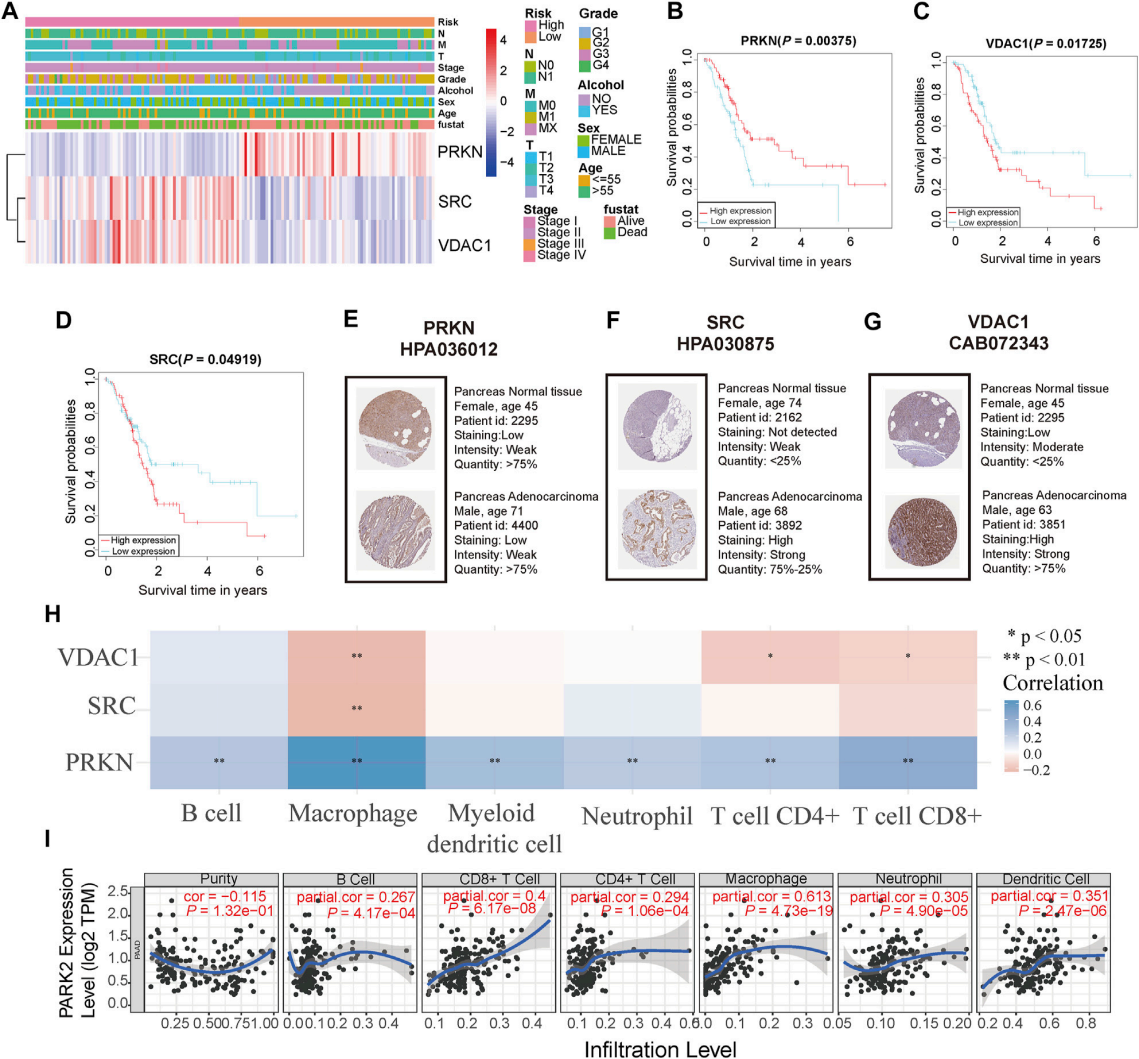
Analyses of the three signature genes **(A)** Heatmap of the expression level of three signature genes and correlation between risk score and clinical features in the TCGA cohort **(B,C,D)** Survival analysis of three signature genes in the TCGA cohort **(E,F,G)** Immunohistochemistry staining of three signature genes in the pancreas normal tissue and pancreas cancer tissue **(H)** The relationship between three signature genes and six immune cells. * represents *p* < 0.05; ** represents *p* < 0.01; *** represents *p* < 0.001 **(I)** PRKN correlates with tumor purity and is significantly positively associated with six immune infiltrates using the TIMER database.

To evaluate the prognostic ability and immune correlations of three signature genes, we assessed their expression level in the TCGA cohort and human immunohistochemical tissue, while survival and immune correlation analyses were performed on these genes. The expression of three signature genes and the correlation between the risk score and clinical features from the TCGA cohort (Age, sex, alcohol, grade, stage, TNM, and status) were presented in Figure 5A. The expression level of PRKN was lowly expressed in the high-risk group compared to the low-risk group, whereas the opposite was observed for SRC and VDAC1 expression. The survival analyses of the three signature genes also showed their tight association with prognosis (Figures 5B–D). Moreover, the expression of signature genes was validated in the tumor samples and normal samples in HPA database. As shown in Figures 5E–G, PRKN was lowly expressed in PC tissue, and SRC and VDAC1 were highly expressed in PC tissue, consistent with the results of gene expression analysis. Next, we further analyzed the relationship between the three signature genes and immune activity, and PRKN was found to be strongly associated with six immune cells (B cell, macrophage, myeloid dendritic cell, neutrophil, CD4^+^ T cell, and CD8^+^ T cell) (all *p* < 0.05, Figure 5H). The result extracted from the TIMER database demonstrated that PRKN was positively correlated with the six immune infiltrating cells **(** Figure 5I).

### Correlation Between Prognostic Model and Clinical Features

**FIGURE 6:**
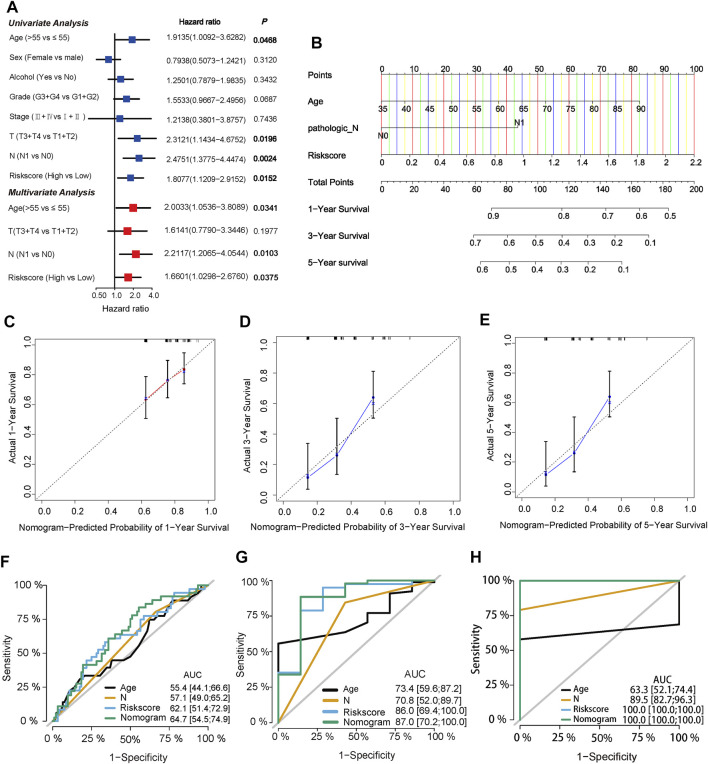
Identification of independent prognostic factors and construction of a predictive nomogram **(A)** Univariate Cox regression analysis and multivariate Cox regression analysis of risk score and clinical characteristics in TCGA cohort **(B)** Nomogram of the TCGA cohort predicting the survival rate at 1, 3, 5-years for PC patients **(C,D,E)** Calibration plots for the nomogram **(F,G,H)** ROC curve analysis of the three independent prognostic factors and nomogram.

To evaluate significant prognostic factors and clinical applicability of the prognostic model, univariate and multivariate analyses were performed to determine the independent prognostic factors, and a nomogram was developed. Clinical information of PC patients, including age, sex, alcohol, grade, stage, T, and N, were extracted from the TCGA cohort. After univariate and multivariate Cox regression analyses, age, N stage, and risk score were identified as independent prognostic factors (*p* = 0.0341, 0.0103, and 0.0375, respectively, Figure 6A). A predictive nomogram was constructed to predict 1-, 3-, five- year survival rates of PC cases based on the independent prognostic factor (Figure 6B). Calibration plots showed a good agreement between the predicted and actual outcomes (Figures 6C–E). The AUC of the nomogram for predicting 1-year, 3-years, 5-years survival was 0.647, 0.870, and 1.00, respectively (Figures 6F–H).

### Functional Enrichment of the Three-Gene Signature

**FIGURE 7:**
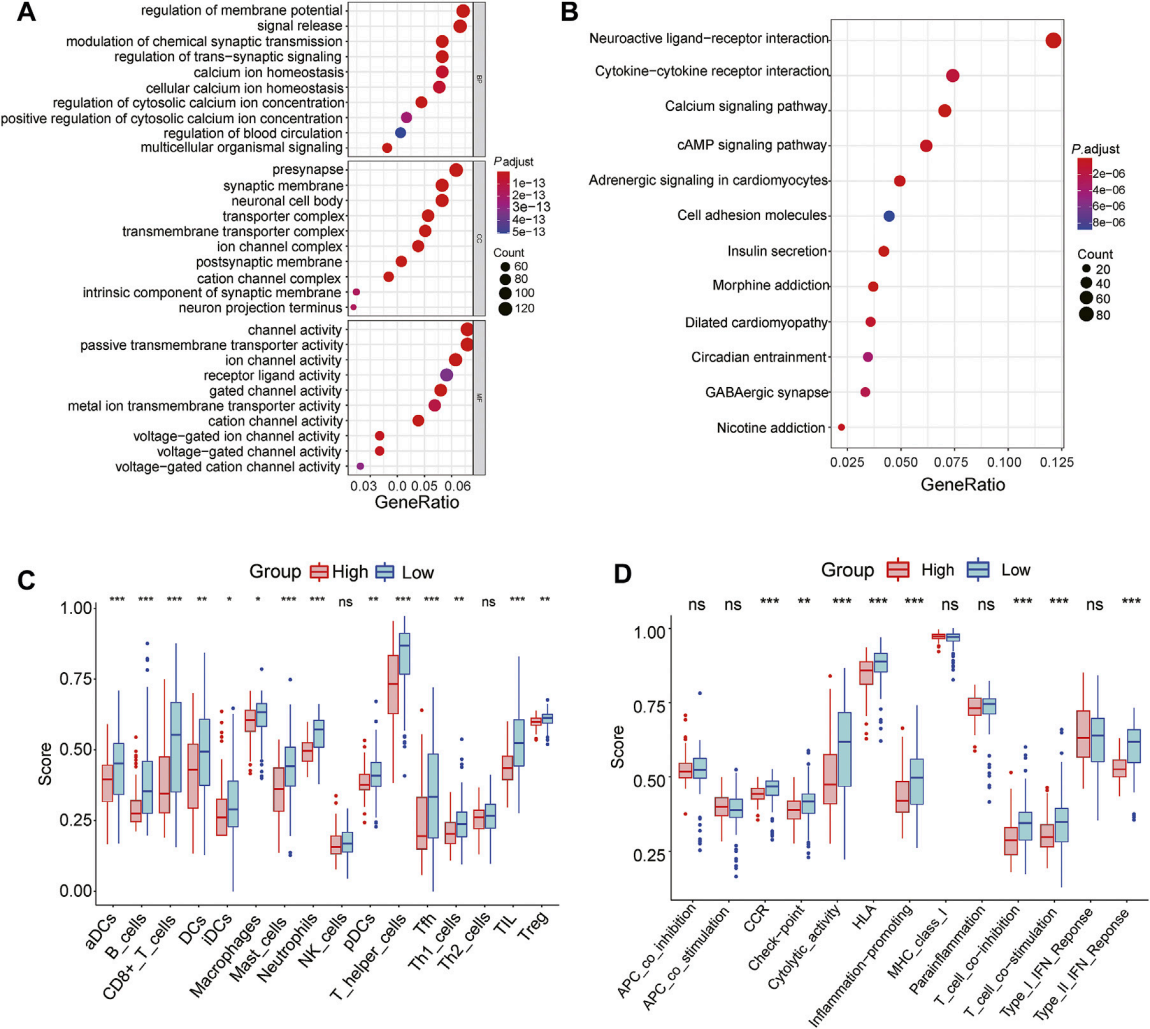
Functional analyses of the 3-gene signature in the TCGA cohort **(A)** GO enrichment analysis of DEGs between the high and low-risk group, BP: Biological process; MF: Molecular function; CC: Cellular components **(B)** KEGG enrichment analysis of DEGs between the high-risk group and low-risk group **(C)** The difference in the enrichment scores of 16 types of immune cells between two risk subgroups **(D)** The difference of enrichment scores of 13 types of immune pathways between two risk subgroups. * represents *p* < 0.05; ** represents *p* < 0.01; *** represents *p* < 0.001.

To further explore the function enrichment of the 3-gene signature, we preformed the GO enrichment, KEGG analysis, and immune enrichment scores on these DEGs between the high and low-risk groups. A total of 2,128 DEGs were screened between two groups in the TCGA cohort. GO enrichment results showed that these DEGs were mainly enriched in regulation of membrane potential and signal release (Figure 7A). KEGG analysis showed significant enrichment of neuroactive ligand-receptor interaction and cytokine-cytokine receptor interaction (Figure 7B). Further functional analysis of the prognostic model demonstrated that the 3-gene signature was significantly associated with immune activity. Most immune cell infiltrating cells in the high-risk group were significantly lower than in the low-risk group (14/16, 87.5%, all *p* < 0.05, Figure 7C). The enrichment scores for immune pathways, such as CCR, check-point, cytolytic activity, HLA, inflammation-promoting, T cell co-inhibition, T cell co-stimulation, Type II IFN Response, were significantly decreased in the high-risk group compared to the low-risk group (all *p* < 0.05, Figure 7D).

### The Three-Gene Signature Was Associated With Immune Microenvironment and Immune Checkpoints

**FIGURE 8:**
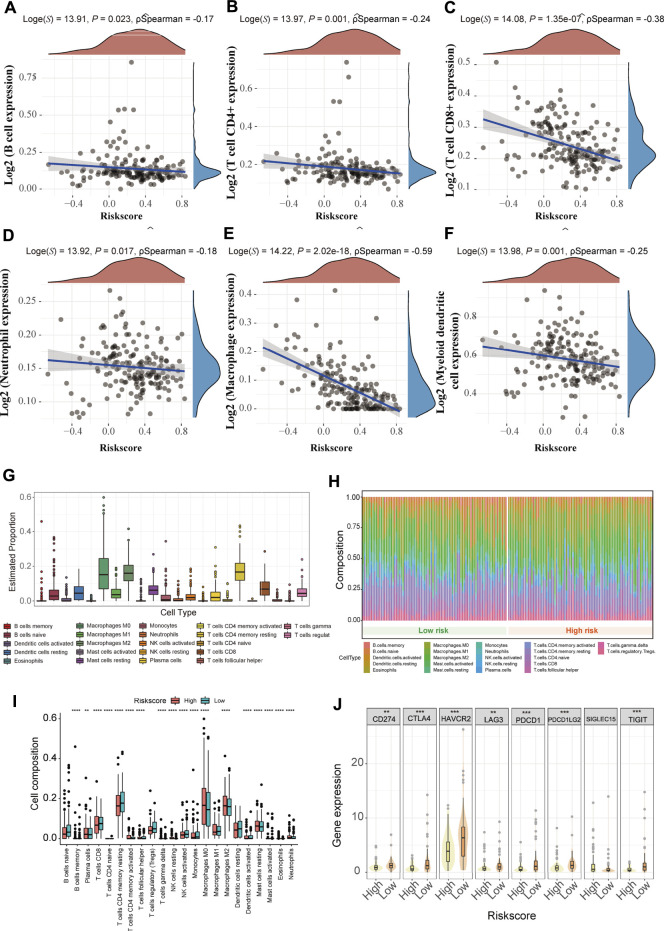
Immune landscape and immune therapy prediction of the 3-gene signature in the TCGA cohort **(A-F)** The correlation between risk score and six immune infiltrating cells (B cells, CD4^+^ T cells, CD8^+^ T cells, Neutrophils, Macrophages, and Dendritic cells) **(G)** The proportion of 22 types of immune cells in the total PC samples **(H)** There are relative proportions of 22 types of immune cells between the high and low-risk subgroup **(I)** The difference in composition of the 22 types of immune cells between two risk subgroups. * represents *p* < 0.05; ** represents *p* < 0.01; *** represents *p* < 0.001 **(J)** The expression level of eight immune checkpoint family genes between two risk subgroups.

To further explore the difference between the 3-gene signature and the immune microenvironment, spearman correlations of risk score and immune score were performed. As shown in Figures 8A–F, the risk score was negatively correlated with six immune infiltrating cells (B cells, CD4^+^ T cells, CD8^+^ T cells, neutrophils, macrophages, and myeloid dendritic cell) (all *p* < 0.05). The proportion of the 22 immune cells in PC patients from the TCGA cohort was presented in Figure 8G. The macrophages M0, macrophages M2, and resting memory CD4^+^ T cells accounted for the highest proportion. The distribution of the 22 tumor-immune cells between the high and low-risk patients is shown in Figure 8H. Specifically, most immune cell infiltrating cells (18/22, 81.8%) were significantly different between the two risk subgroups (all *p* < 0.05), indicating a strong correlation between the 3-gene signature and the immune microenvironment (Figure 8I). In addition, we investigated the correlation between the prognostic model and the expression values of immune checkpoints genes, which can serve as an indicator to predict the immune response. As shown in Figure 8J, except for the SIGLEC15 gene, the other seven immune checkpoints genes were downregulated in the high-risk patients from the TCGA cohort (all *p* < 0.05).

### The Mutation Landscape of the Three-Gene Signature

**FIGURE 9:**
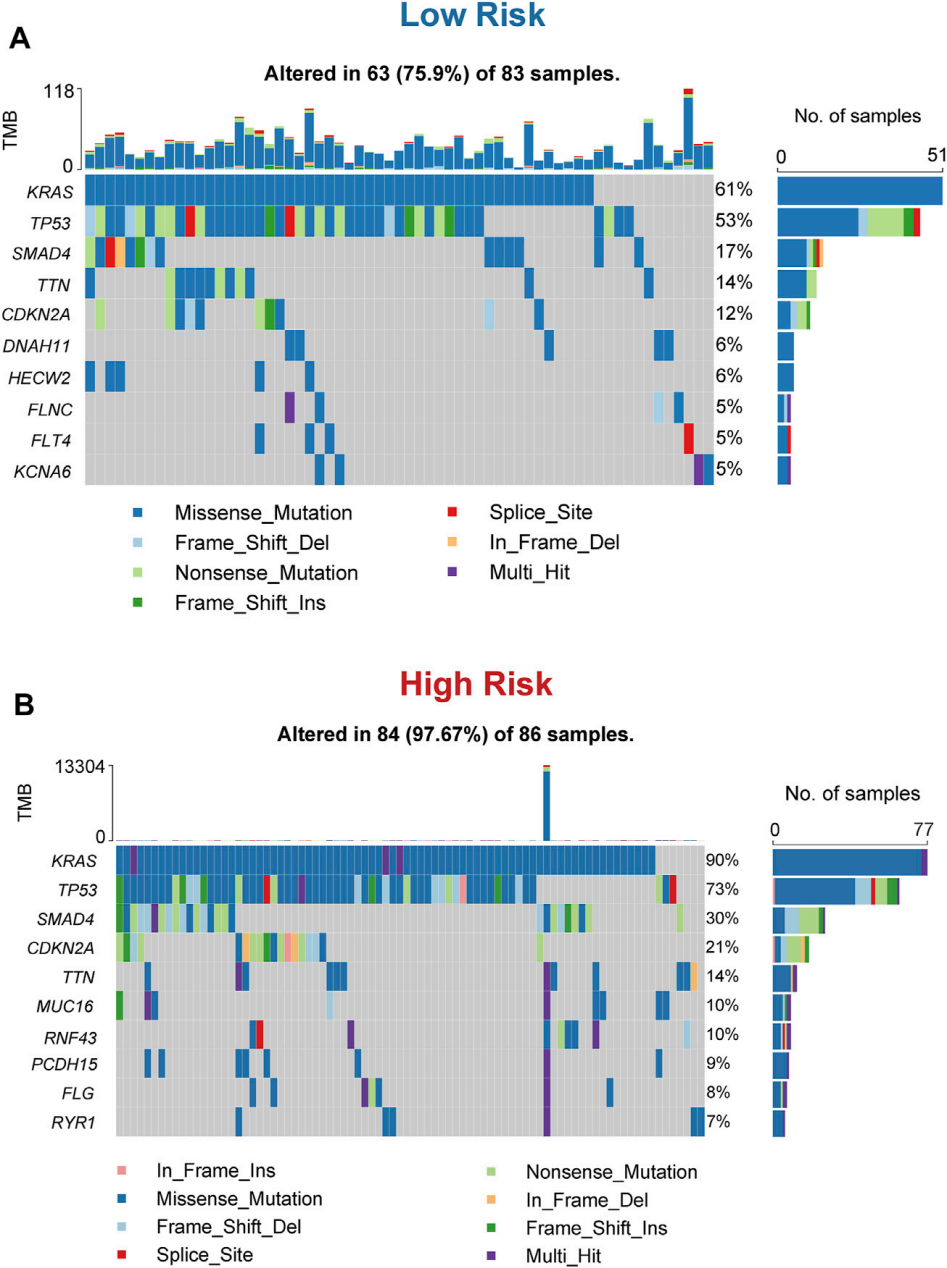
Mutational landscape of the 3-gene signature **(A)** Waterfall plot of mutation status in the low-risk patients **(B)** Waterfall plot of mutation status in the high-risk patients.

It has been established that somatic hypermutations are a characteristic of PC, and thus, we compared the mutation landscapes between the high and low-risk patients in the TCGA cohort. As shown in Figures 9A,B, a higher mutation rate was observed in high-risk patients than in low-risk patients (97.67 vs 75.9%). Regarding the gene mutation frequency, KRAS, tumor protein p53 (TP53), and SMAD family member 4 (SMAD4) were the most altered gene in high-risk patients than in low-risk patients (90 vs 61%, 73 vs 53%, and 30 vs 17%, respectively). Moreover, missense mutations were the most common mutation type in PC patients.

### The 3-Gene Signature Can Predict Chemotherapy Drug Sensitive

**FIGURE 10:**
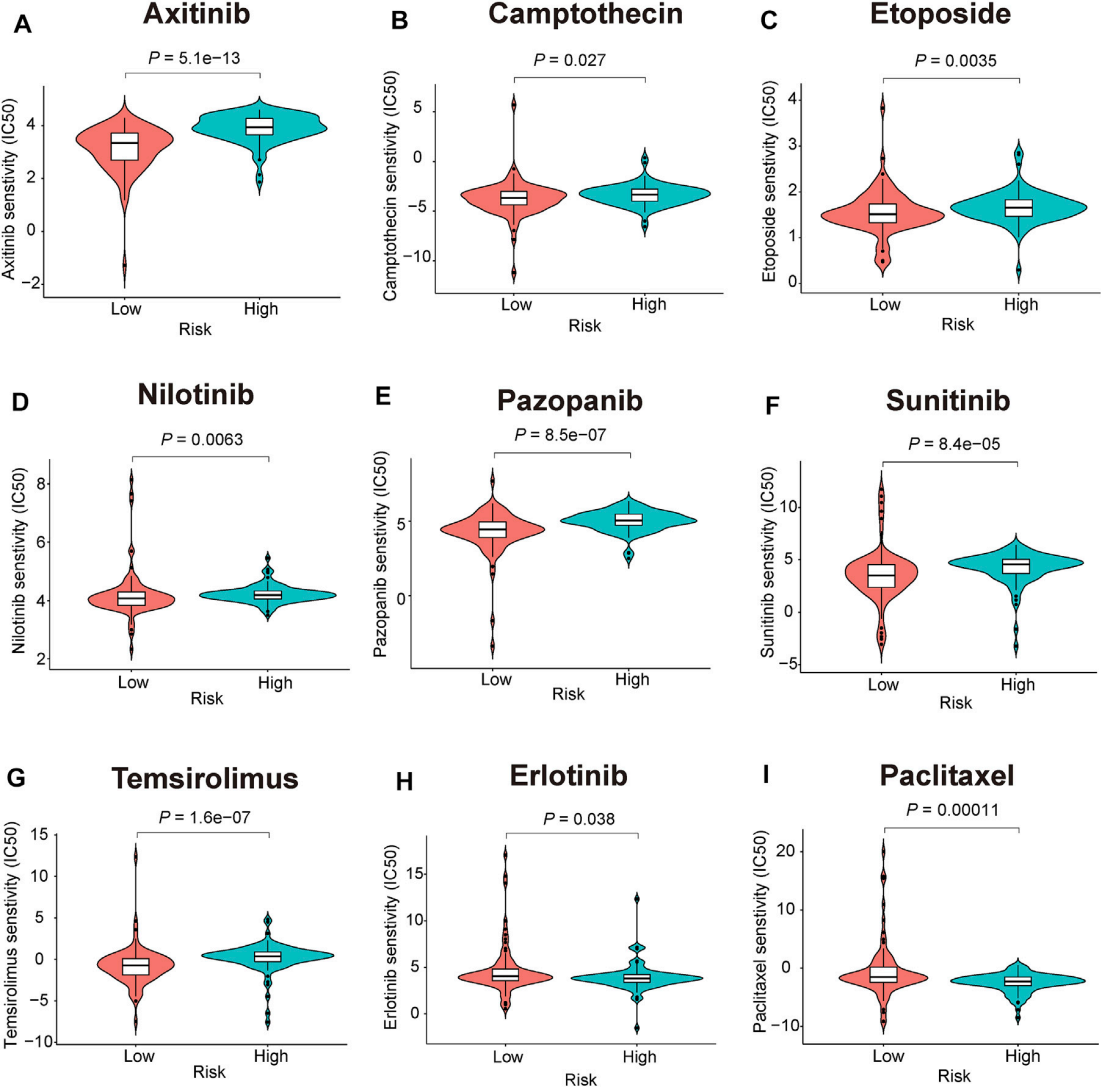
Chemotherapeutic responses prediction of the 3-gene signature **(A-I)** Estimated IC50 for **(A)**, axitinib **(B)**, camptothecin **(C)**, etoposide **(D)**, nilotinib **(E)**, pazopanib **(F)**, sunitinib **(G)**, temsirolimus **(H)**, erlotinib **(I)**, paclitaxel.

Chemotherapy drugs have remained the mainstay for the treatment of PC. Poor prognosis has been associated with chemoresistance. Herein, we further predicted the chemotherapy response of the two risk subgroups to common chemotherapy drugs. As shown in Figures 10A–G, high-risk patients had higher estimated IC50s for seven chemotherapy drugs (axitinib, camptothecin, etoposide, nilotinib, pazopanib, sunitinib, and temsirolimus) than patients in the low-risk group, indicating that low-risk patients can benefit from the chemotherapy agents. In addition, we found that high-risk patients were more sensitive to erlotinib and paclitaxel (*p* = 0.038, *p* = 0.00011, respectively, Figures 10H,I).

## Discussion

Mitophagy is a key mechanism for metabolic reprogramming and aerobic glycolysis regulation within tumor cells, and has huge prospects as a novel approach for treating various cancers (Xie et al., 2021). Mitophagy has a dual role in PC progression, which mainly depends on their clinical features, tumor microenvironment, and mutation status (Li et al., 2018; Humpton et al., 2019). For example, PINK1/PRKN mediated mitophagy inhibition may promote pancreatic tumorigenesis (Li et al., 2018), whereas the loss of BCL2 interacting protein three like (BNIP3L) may suppress KRAS-driven pancreatic tumorigenesis (Humpton et al., 2019). Although mitophagy’s role in pancreatic tumorigenesis has been well documented, the extent to which mitophagy influences prognosis and the therapeutic strategy in PC remains unclear. In this study, we conducted a comprehensive analysis of the value of mitophagy-related genes in conducting risk stratification, immune activity, genetic mutation, and chemotherapy response of PC patients. We found that consensus mitophagy-related genes could serve as a PC classifier. Our 3-gene signature based on immunoscore classification was developed to stratify PC patients. Patients in the high-risk groups were associated with poor survival, lower immune infiltration level, higher mutation load, and poor therapeutic sensitivity.

Our prognostic model consisted of three mitophagy-related genes (PRKN, SRC, VDAC1), and the combination of these three genes yielded good performance during clinical prognosis assessment. PRKN, also named PARK2, has been documented to function as an inhibitor of pancreatic tumorigenesis by suppressing inflammation-related immunosuppression (Kang et al., 2019). Consistent with our study finding, low PRKN expression has been correlated with poor prognosis in PC patients (Li et al., 2018). In addition, we found that PRKN was tightly associated with six immune infiltrating cells, which accounted for their role in anti-tumor immunity. SRC is a non-receptor tyrosine kinase responsible for tumor cell apoptosis, migration, and transformation (Fianco et al., 2016). SRC has been reported to be highly expressed in PC samples (Castillo et al., 2020), and activation of SRC contributes to PC tumorigenicity (Ahn et al., 2018). VDAC1 mainly participated in cell volume regulation and apoptosis (Shoshan-Barmatz et al., 2020). It has been reported that VDAC1 mRNA and protein are highly expressed in PC tissues (Kuo et al., 2016) and loss of VDAC1 significantly suppressed cell growth, invasion and migration in the PC cell (Wang et al., 2016). Accordingly, these three mitophagy-related genes play a vital role during PC tumorigenesis and might have clinical value as potential biomarkers.

Further analyses showed high predictive accuracy and robustness of the mitophagy-related gene signature in this study. Moreover, patients in the high-risk groups had worse outcome in the training and validation cohorts. The 3-years survival AUC value was 0.785 in the TCGA cohort, 0.818 in the ICGC cohort, 0.821 in the GSE28735, and 0.822 in the GSE62452, validating the model’s excellent accuracy compared to other prognostic models reported in the literature (AUC = 0.698, (Ma et al., 2021); AUC = 0.657 (Yuan et al., 2021)). After univariate and multivariate Cox regression analyses were conducted, the risk score was identified as an independent prognosis factor. Next, we constructed a quantitative and objective nomogram based on multivariate analysis. The AUC value demonstrated that our nomogram was significantly better than clinical staging, appropriate for use during clinical practice.

The tumor microenvironment, especially the immune microenvironment, plays a vital role during pancreatic cancer relapse and metastasis, limiting the treatment efficacy of immunotherapy and chemotherapy (Ren et al., 2018; Zhang X. et al., 2020). Accumulating research on tumor microenvironment has highlighted the vital function of immune cell infiltration in PC progression, metastasis, and immune escape (Shiau et al., 2017; Shen et al., 2018; Chen et al., 2021). In the present study, the mitophagy-related signature was significantly associated with the infiltration of immune cells. For example, the high-risk group was infiltrated by a higher proportion of macrophages M0, macrophages M2, resting NK cells and low proportions of CD8^+^T cells and CD4^+^T cells. Tumor-associated macrophages (TAMs) might function as promoter during tumor progression (De Palma and Lewis, 2013). Generally, TAM composed of multiple subpopulations macrophages including M0 (inactivated macrophages), M1 (classically activated), and M2 (alternatively activated) phenotypes (Locati et al., 2020). Polarizing from M0 macrophages, M2 cells contribute to immunosuppression and angiogenesis by generating immunosuppressive factors such as interleukin-10 (Gong et al., 2020). NK cells are a type of lymphocyte that are involved in tumor immunity, which can be divided into resting and activated subtypes (Wu et al., 2020). Typically, the higher the proportion of resting NK cells is, the tumor-infiltrating level will be, which is conducive to formation of tumor microenvironment (Newman et al., 2015). In addition, CD8^+^ T cells and CD4^+^ T cells are widely acknowledged as the main components of tumor-infiltrating lymphocytes (TLSs), and participate in anti-tumor immunity (Farhood et al., 2019; Miller et al., 2021). In the pancreatic tumor microenvironment, CD8^+^ T cells were found to blockade PD-1 checkpoints and CXCR4 to exert an antitumor effect, suggested the promising objectives of immunotherapies (Seo et al., 2019). Typically, immune activation can be increased by blockading immune checkpoints, and thus enhanced immune checkpoint gene expression is considered as indicator to promote response to immunotherapy in clinical practice (Sehgal et al., 2015). In our study, we further explored the expression of eight immune checkpoint genes (CD274, CTLA4, HAVCR2, LAG3, PDCD1, PDCD1LG2, SIGLEC15, TIGIT) between two risk subtypes and the results showed elevated expression of seven immune checkpoint genes in low-risk patients, indicating that patients in the low-risk group could benefit from immunotherapies by blocking immune checkpoints. Higher immunosuppressive cell infiltration and lower expression of immune checkpoints in high-risk PC patients may in turn promote resistance to immunotherapy, suggested that the poor prognosis of high-risk patients was correlated with the induction of an immunosuppressive microenvironment.

In cancer risk assessment, mutations identification is also a key step, which may contribute to develop preventive or therapeutic strategies in PC. In our study, we further explore the mutation landscape of mitophagy-related gene signature. High-risk group exhibited more somatic mutation count. Among them, the incidence of KRAS mutation in the high-risk group was strongly higher than the low-risk group among them (90 vs 61%). Mutations in KRAS are related to multiple cancers, especially in PC, and oncogenic KRAS mutations can drive PC Metastasis through Mutant p53 (Kim et al., 2021). Recently, some researchers proposed that combination of KRAS mutation-based cancer vaccines and other immune therapy methods may evade immunosuppression and enhance the chance of a successful tumor treatment (Zhang Y. et al., 2020). Identification of KRAS mutational status is significant to manage these patients as the high-risk patients of PC may benefit from KRAS-driven therapeutic cancer vaccines.

Another major obstacle encountered during PC treatment is chemotherapy resistance that plays an important role in cancer mortality. Distinction of chemosensitive population may maximize the anti-tumor effects in PC patients that can benefit from standard chemotherapeutic regimens. Our study performed a chemotherapeutic sensitivity analysis between mitophagy-related risk subgroups based on GDSC database, and the high-risk patients were more sensitive to erlotinib and paclitaxel. A multicentre study supports the anti-tumor activity of the nab-paclitaxel combination of gemcitabine for locally advanced PC patients (Philip et al., 2020). In addition, gemcitabine/erlotinib plus oxaliplatin showed higher response rates and improved progression-free survival in locally advanced or metastatic PC patients (Lim et al., 2021). In contrast, the low-risk patients were more sensitive to axitinib, camptothecin, etoposide, nilotinib, pazopanib, sunitinib, and temsirolimus. This finding provides valuable information to oncologists during drug selection. Nevertheless, the exact mechanism of the indicated agents during PC treatment warrants further studies and experimental validation.

In summary, we developed and validated a mitophagy-related gene signature for prognostic stratification in PC patients. Moreover, the novel signature could be applied for survival and therapeutic response prediction during the treatment of PC patients.

## Data Availability

All data are available in the TCGA database (https://portal.gdc.cancer.gov/repository), ICGC database (https://dcc.icgc.org), GEO database (https://www.ncbi.nlm.nih.gov/geo), ESTIMATE database (https://bioinformatics.mdanderson.org/estimate/disease.html), and Pathway Unification database (https://pathcards.genecards.org/).
